# Quercetin Regulates Calcium and Phosphorus Metabolism Through the Wnt Signaling Pathway in Broilers

**DOI:** 10.3389/fvets.2021.786519

**Published:** 2022-01-27

**Authors:** Bo Wang, Shanshan Wang, Manyi Ding, Han Lu, Hao Wu, Yao Li

**Affiliations:** Institute of Animal Nutrition, College of Animal Science and Technology, Northeast Agricultural University, Harbin, China

**Keywords:** quercetin, broiler, calcium and phosphorus metabolism, Wnt signaling pathway, transcriptomics

## Abstract

This study intended to explore the effect and mechanism of different doses of dietary quercetin on calcium and phosphorus metabolism to provide an experimental basis for preventing leg disease in broilers. A total of 480 1-day-old healthy Arbor Acre broilers were randomly allotted into four groups (0, 0.02, 0.04, 0.06%) for 42 days. Compared with control, 0.06% quercetin significantly increased the unit weight and the relative weight of tibia in broilers (*P* < 0.05). Meanwhile, phosphorus content and bone mineral density (BMD) were significantly increased by 0.06% dietary quercetin supplementation in tibia (*P* < 0.05). Ash of tibia was significantly increased by 0.04 and 0.06% quercetin in broilers (*P* < 0.05). In addition, 0.06% quercetin significantly increased the content of serum calcium-binding protein (CB), estradiol (E_2_), osteocalcin (OC), alkaline phosphatase (ALP), and calcitonin (CT) (*P* < 0.05); 0.04% quercetin significantly increased 1,25-dihydroxyvitamin D_3_ (1,25-(OH)_2_D_3_) (*P* < 0.05) content in serum of broilers. The content of serum parathyroid (PTH) was significantly decreased by 0.02 and 0.06% quercetin (*P* < 0.05) in broilers. Gene Ontology (GO) functional annotation and Kyoto Encyclopedia of Genes and Genomes (KEGG) enrichment analysis showed that the Wnt signaling pathway was a key signaling pathway of calcium and phosphorus metabolism in broilers which was significantly regulated by quercetin. The differentially expressed genes (DEGs) from transcriptome sequencing were validated with real-time quantitative PCR (RT-qPCR). In conclusion, 0.06% dietary quercetin supplementation improved calcium and phosphorus metabolism by regulating the Wnt signaling pathway in broilers.

## Introduction

The poultry industry is vital for creating the national economy. Poultry-provided animal protein is widely consumed by humans, namely chicken and eggs. Poultry was the largest source of protein worldwide in 2019 (1). However, intensive production has increased the level of modern broiler production, and has also greatly increased the incidence of leg disease in broilers (2, 3). Tibial dyschondroplasia (TD) is a common bone disease in rapidly growing poultry throughout the world that causes movement and standing difficulties in broilers (4). In modern feeding patterns, broilers grow faster but are at higher risk of TD due to fast movement and weight gain, resulting in weak bones and lameness, and the disease incidence is as high as 40–60% in the flocks (5, 6). Therefore, leg diseases are considered to be the biggest economic threats to the poultry industry and may not be ignored in the breeding industry of broilers (7, 8). The production cycle of chickens is short, most drugs with serious drug residues have a long metabolic cycle, and the treatment cost is high, which severely restricts the initiative of TD treatment (9). In the modern breeding industry, the deficiency or low utilization rate of calcium and phosphorus in feed or the imbalance of calcium and phosphorus may further induce bone malnutrition in broilers. The balance between calcium and phosphorus in the feed is vital to leg health of broilers (10–12). To effectively improve the metabolism of calcium and phosphorus and reduce the occurrence of TD in broilers, functional feed additives to prevent leg disease in broilers need to be studied (13, 14).

Flavonoids have estrogen-like effects (15). Estrogen may promote the intestinal absorption of calcium (16), and flavonoids cooperate with estrogen to affect calcium and phosphorus metabolism. Quercetin, a type of flavonoid, is abundant in various tea, fruits, and leaves, and has antioxidant, anti-inflammatory, anti-allergic, antibacterial, and antiviral activities (17–19). Therefore, rational use of quercetin will produce huge economic benefit. In addition, quercetin may improve the absorption of calcium in the small intestine, and enhance the activity of vitamin D receptor (VDR) (20). One study confirmed the positive effects of proanthocyanidins and cannabinoids on bone health, which improved bone weight loss, bone length and diameter, content of calcium and phosphorus in ashes, and bone mineral density (BMD) in rats (21). VDR expression of bone cells was low in ovariectomized rats, after estrogen treatment, VDR expression was significantly increased, this implied that estrogen may enhance VDR expression in bone (22). It suggested that quercetin may also promote calcium absorption in the small intestine, thereby regulating bone calcium metabolism. However, the specific mechanism of action remains unclear.

The intestine plays a major role in the digestion and absorption of calcium and phosphorus. Ca^2+^ was mainly absorbed in the small intestine, especially in the duodenum (23, 24). 1,25-dihydroxyvitamin D_3_ (1,25-(OH)_2_D_3_) combined with parathyroid (PTH) and calcitonin (CT) forms the most important hormone in the body that maintains calcium homeostasis (25). Absorption of calcium in the small intestine by 1,25-(OH)_2_D_3_ was mainly concentrated in the transcellular transport pathway and bound to specific receptors in the small intestinal mucosa, and increased the synthesis of calcium-binding protein (CB), thus promoting Ca^2+^ absorption (26, 27). Flavonoids were mainly absorbed and utilized in the small intestine, while the absorption of calcium in the intestine mainly depends on hormones such as CB, 1,25-(OH)_2_D_3_, and PTH. Then, hormones may be a potential target for flavonoids to regulate calcium and phosphorus metabolism. The calcium and phosphorus metabolism was mainly transported in the duodenum. However, no examination was done on the mechanism of quercetin regulating calcium and phosphorus metabolism in specific intestinal segments, and quercetin regulating calcium and phosphorus metabolism at the molecular level in duodenum of broilers has not been reported.

The aim of this study was to evaluate the mechanism of quercetin regulating calcium and phosphorus metabolism in broilers. Therefore, the effects of quercetin on tibia development, calcium deposition, serum biochemical index, and transcriptome changes of duodenal mucosa in broilers were studied to elucidate the mechanism of dietary quercetin supplementation regulating calcium and phosphorus metabolism. The results of this experiment will provide the scientific basis for researching the use of quercetin in practical production to improve broiler tibia development in the future.

## Materials and Methods

### Animal and Diets

A total of 480 (1-day-old) healthy Arbor Acre broilers (similar in body weight, 47.17 ± 1.26 g) were obtained from a commercial company (Yinong Poultry, Harbin, China). The chickens were randomly divided into four experimental groups with six replicates per group and 20 broilers per replicate.

Broilers were raised in a netted stainless steel cage (526 × 423 × 381 mm) with *ad libitum* access to feed and fresh water, and controlled ventilation. All chickens experienced 16 h of continuous light every day for 42 days. Temperature was maintained at 32–34°C for the first 3 days and decreased to 24°C by the end.

The experimental diets were formulated based on corn and soybean meal according to Chinese Broiler Feeding Standards (GB/T5916-2020) to meet the nutrient requirements of broilers (Table 1). And four concentrations of quercetin were added to the diet: 0.00, 0.02, 0.04, and 0.06%. Feeding was divided into two phases: the starter phase from 1 to 21 days and the grower phase from 22 to 42 days. Quercetin dihydrate powder with 97% purity (Sigma-Aldrich, United States) was mixed in basal diet.

**Table 1:** Analysis composition of basal diets and nutrient level (air dry basis, %).

**Items**	**Content (1–21days)**	**Content (22–42 days)**
**Ingredients**
Corn	58.00	62.50
Soybean meal	34.00	29.80
Soybean oil	3.00	3.00
Fish meal	1.00	1.00
Methionine	0.20	0.20
Dicalcium phosphate	1.58	1.75
Limestone	1.54	1.12
sodium chloride	0.35	0.30
Multivitamin Premix^a^	0.03	0.03
Mineral Premix^a^	0.20	0.20
Choline	0.10	0.10
Total	100.00	100.00
**Nutrient level^b^**
Metabolic energy (ME) (MJ/kg)	12.39	12.57
CP	20.32	18.83
Lys	1.09	0.99
Met + Cys	0.64	0.60
Ca	1.10	0.98
Total *P*	0.68	0.70
Available *P*	0.40	0.43

a *Amount provided per kilogram of diet: vitamin A = 1,500 IU; vitamin D_3_ = 3,200 IU; vitamin E = 10 IU; vitamin K = 0.5 mg; vitamin B_1_ = 1.8 mg; vitamin B_2_ = 3.6 mg; vitamin B_6_ = 3.5 mg; vitamin B_12_ = 0.01 mg; biotin = 0.15 mg; folic acid = 0.55 mg; niacin = 30 mg; pantothenic acid = 10 mg; Cu (CuSO_4_·5H_2_O) = 8 mg; I (KI) = 0.35 mg; Fe (FeSO_4_·7H_2_O) = 80 mg; Mn (MnSO_4_·H_2_O) = 60 mg; Se (NaSeO_3_) = 0.15 mg; Zn (ZnO) = 40 mg*.

b *The values were calculated based on dry matter basis*.

### Sample Collection and Preparations

At 42 days of age, broilers were randomly slaughtered from six replicates in four groups. After 12 h of fasting, broilers were weighed and euthanized by cervical dislocation. The jugular vein blood samples (10 ml) were collected and placed on ice. The tibia was quickly separated, the adherent tissue was removed, then cleaned with saline, and stored at −20°C for further analysis. About 5 g of duodenal mucosa was placed in liquid nitrogen and then preserved at −80°C. All procedures used in this study were approved by the Animal Welfare Committee of Northeast Agricultural University (Harbin, China). Housing, management, and care of the birds were carried out in accordance with the guidelines for the Agricultural Animal in Agricultural Research and Teaching of Heilongjiang Province (HEI Animal Management Certificate No. 11928).

### Tibia Index

The tibial length was measured with a vernier caliper, the wet weight was weighed by electronic balance, and the unit weight and relative weight of tibia were calculated.

Unit weight of tibia (g/cm) = fresh weight of tibia (g)/length of tibia (cm).

Relative tibia weight (%) = [weight of tibia (g)]/[weight of broiler (g)] × 100

### Calcium and Phosphorus Deposition

Calcium concentration in tibia was determined by an atomic absorption spectrophotometer, and P was determined by the vanadium molybdate colorimetric method. The ash content of tibia was expressed as ash per unit tibial length, and expressed as a percentage of dry weight (28). BMD in tibia was measured using a Qdr-4500w dual energy X-ray bone mineral density detector.

### Serum Biochemical Index

Serum biochemical indices were assessed according to the content of alkaline phosphatase (ALP), calcium (Ca), phosphorus (P), osteocalcin (OC), estradiol (E_2_), CB, PTH, 1,25-(OH)_2_D_3_, and CT by an ELISA assay kit (Nanjing Jiancheng Bioengineering Institute, Nanjing, Jiangsu, P. R. China).

### High-Throughput RNA Sequencing

In this endeavor, 12 samples of duodenal mucosa were frozen in dry ice and sent to Genesis (Beijing) Co. Ltd. for sequencing using the HiSeq 2000 System (Illumina, Inc, USA). The results were compared with the database and the markers of each gene were prepared for the subsequent experimental analysis.

### Real-Time Quantitative PCR

The mRNA expression of genes in the duodenal mucosa of broilers was determined using real-time quantitative polymerase chain reaction (RT-qPCR) (7500 Real-Time PCR System, Singapore). Primers were designed using Primer 5.0 software based on mRNA sequences of broiler genes published on GenBank, and Primers were synthesized (Sangon Biological Co. Ltd., Shanghai, China). The sequences of primers are showed in Table 2. After samples from the frozen duodenal mucosa were ground into fine powder in a liquid nitrogen environment, total RNA was extracted using the TRIZOL (TaKaRa, Japan) reagent. Genomic DNA was removed from total RNAs by the DNA-free kit (TaKaRa Biotechnology, Co., Ltd. Dalian, China). The integrity of RNA was detected on 1.5% agarose gel and shown by ethidium bromide staining. The 260/280 nm absorbance ratio of all RNA samples of duodenal mucosa was between 1.8 and 2.2. First strand cDNA was synthesized from 2 μg total RNA by RNA reverse transcription. The reaction mixtures were incubated in the following conditions: reverse transcription at 37°C for 15 min and inactivation of reverse transcriptase at 85°C for 5 min, until the temperature was decreased to 4°C. Reverse transcription was performed to create cDNA (2 μl) from the total RNA to use as a template for RT-qPCR. The cDNA was amplified by PCR in 20 μl of reaction mixture. The RT-qPCR reaction was carried out according to the instructions of the SYBR Real-Time PCR kit (TaKaRa Biotechnology, Co., Ltd. Dalian, China). The PCR condition was as follows: initial denaturation at 95°C for 30 s, followed by PCR reaction at 40 cycles of 95°C for 5 s and 60°C for 34 s, and melting curve analysis at 95°C for 15 s, 60°C for 2 s, and 95°C for 15 s. The specificity of the qPCR reaction was monitored by melting curve analysis and gel electrophoresis. β-actin was used as the internal control in this study (Table 2). All experiments were repeated in triplicate. The data were analyzed with the comparative cycle threshold method (2^−ΔΔCT^) in Microsoft Excel software.

**Table 2:** Primers of genes used for mRNA expression level.

**Genes**	**Primers**	**Sequence (5^**′**^ → 3^**′**^)**	**Product size**	**GenBank accession**
Wnt-5a	F	ATGGACGGCTGTGAACTGATGTG	102 bp	XM_015292954.2
	R	CACGTAGCAGCACCAGTGGAAC		
CAMK2G	F	TCGAAGAACAGCAAGCCGATACAC	200 bp	XM_015288320.2
	R	GAGCAGTGGTAGTGGACATTCAGC		
CAMK2D	F	TCACCGACGAGTACCAGCTCTTC	99 bp	XM_015276289.2
	R	TGGCAGCATACTCCTGTCCTGTG		
CAMK2B	F	CCGAAGCCAAGAACCTCATCAACC	150 bp	XM_025142761.1
	R	TCTTCAGGCACTCCACCGTCTC		
PLCB4	F	GTGCTGACCAGGAAGAAGAAGCTC	149 bp	NM_001199435.1
	R	ATACGATGCCATCCATGCCTGTTC		
PRKCA	F	GTGATGCTGGCGGACAGGAAG	157 bp	XM_025141605.1
	R	AGTGAAGCTGTGTCAGGAATGGTG		
NFATC1	F	CGGATACGGAGGACACATTGACA	198 bp	XM_025147635.1
	R	GCAGTGGAAGGTGATCGCTTGG-		
β-Actin	F	GAGAAATTGTGCGTGACATCA	152 bp	NM_205518.1
	R	CCTGAACCTCTCATTGCCA		

### Statistical Analysis

All data from this experiment were analyzed by one-way ANOVA as a completely randomized design with four treatments and six replicates for each treatment using SPSS 21.0 statistical software (SPSS Inc., Chicago, IL). All the results were expressed as the “mean values ± standard deviation”. Calculated ΔCt (corrected sample) = mean value of target gene − mean value of internal reference gene, ΔΔCt = ΔCt-mean value of control group. Differences with treatment means with a possibility of *P* < 0.05 were considered as statistically significant.

## Results

### Effect of Quercetin on Tibia Development in Broilers

The unit weight of tibia was significantly increased with increasing quercetin (*P* < 0.05). The relative weight of tibia was significantly increased by 0.06% quercetin (*P* < 0.05). Dietary quercetin supplementation at the level of 0.04% also significantly increased the unit weight of tibia (*P* < 0.05). However, quercetin did not affect tibia length (*P* > 0.05), compared with control (Table 3).

**Table 3:** Effect of quercetin on length and weight of tibia in broilers.

**Items**	**Control**	**0.02% quercetin**	**0.04% quercetin**	**0.06% quercetin**	***P***
Relative weight (g/cm)	1.73 ± 0.05^b^	1.77 ± 0.03^ab^	1.77 ± 0.04^ab^	2.33 ± 0.15^a^	0.000
Unit weight (%)	0.89 ± 0.03^b^	1.01 ± 0.03^a^	1.04 ± 0.02^a^	1.12 ± 0.07^a^	0.009
Length (cm)	11.07 ± 0.25	11.71 ± 0.13	11.48 ± 0.36	11.88 ± 0.14	0.121

*In the same row, values with different small letter superscripts mean significant difference P < 0.05; values with no letter or the same letter superscripts mean no significant difference P > 0.05. The data are represented as mean ± SEM, and n = 6 per group*.

### Effect of Quercetin on Calcium and Phosphorus Deposition in Broilers

Compared with control, the content of phosphorus and BMD in tibia was significantly increased by 0.06% quercetin (*P* < 0.05). Dietary quercetin supplementation at levels of 0.04 and 0.06% also significantly increased ash of tibia (*P* < 0.05). The content of calcium in tibia tended to increase (*P* = 0.079) (Table 4).

**Table 4:** Effect of quercetin on content of calcium and phosphorus and bone density in tibia of broilers.

**Items^**a**^**	**Control**	**0.02% quercetin**	**0.04% quercetin**	**0.06% quercetin**	***P***
Tibia phosphorus	5.61 ± 0.06^b^	5.67 ± 0.09^b^	5.82 ± 0.04^b^	6.06 ± 0.09^a^	0.002
BMD	0.29 ± 0.01^b^	0.31 ± 0.01^ab^	0.31 ± 0.00^ab^	0.33 ± 0.10^a^	0.005
Tibia ash	36.92 ± 0.83^b^	38.21 ± 0.20^b^	39.70 ± 0.74^a^	40.17 ± 0.99^a^	0.011
Tibia calcium	12.05 ± 0.40	12.38 ± 0.40	13.04 ± 0.19	13.29 ± 0.37	0.079

*In the same row, values with different small letter superscripts mean significant difference P < 0.05; values with no letter or the same letter superscripts mean no significant difference P > 0.05. The data are represented as mean ± SEM, and n = 6 per group*.

### Effect of Quercetin on Serum Biochemical Indexes of Broilers

Compared with control, the content of serum CB, OC, and ALP in broilers was significantly increased with increasing quercetin (*P* < 0.05). Dietary quercetin supplementation at the level of 0.06% significantly increased the content of serum E_2_ and CT in broilers (*P* < 0.05). The content of serum E_2_ and 1,25-(OH)_2_D_3_ in broilers was significantly increased by 0.04% quercetin (*P* < 0.05). The content of serum of PTH in broilers was significantly decreased by 0.02 and 0.06% quercetin (*P* < 0.05). However, dietary quercetin supplementation did not affect the content of serum P and Ca in broilers (*P* > 0.05) (Table 5).

**Table 5:** Effect of quercetin on serum biochemical parameters in broilers.

**Items**	**Control**	**0.02% quercetin**	**0.04% quercetin**	**0.06% quercetin**	***P***
CB (ng/mL)	6.51 ± 0.19^d^	8.74 ± 0.78^c^	11.88 ± 0.54^b^	14.73 ± 0.66^a^	0.000
E_2_ (pg/mL)	6.33 ± 0.23^b^	6.32 ± 0.74^b^	9.75 ± 0.81^a^	10.60 ± 2.48^a^	0.001
OC (ng/mL)	4.26 ± 0.54^b^	6.78 ± 0.70^a^	7.54 ± 1.47^a^	9.19 ± 0.65^a^	0.010
ALP (U/dL)	10.75 ± 1.02^b^	13.30 ± 0.59^a^	13.60 ± 0.56^a^	14.43 ± 1.04^a^	0.031
PTH (ng/dL)	49.98 ± 2.27^a^	43.17 ± 1.02^b^	46.09 ± 1.38^ab^	44.75 ± 0.67^b^	0.023
CT (pg/mL)	81.50 ± 0.23^b^	82.18 ± 0.76^b^	82.91 ± 0.67^ab^	84.44 ± 0.67^a^	0.019
1,25-(OH)_2_D_3_ (pg/mL)	175.78 ± 25.07^b^	232.72 ± 33.05^ab^	298.95 ± 25.94^a^	229.74 ± 8.81^ab^	0.020
P (mmol/L)	2.00 ± 0.17	2.27 ± 0.17	2.38 ± 0.13	2.43 ± 0.09	0.180
Ca (mmol/L)	2.21 ± 0.20	2.30 ± 0.14	2.38 ± 0.04	2.50 ± 0.12	0.523

*In the same row, values with different small letter superscripts mean significant difference P < 0.05; values with no letter or the same letter superscripts mean no significant difference P > 0.05. The data are represented as mean ± SEM, and n = 6 per group*.

### Overall Assessment for Mapping Statistics

Sequence data from the 12 samples were mapped to the reference genome (Gallus gallus, 5.0). The RNA-seq libraries of the 12 samples were sequenced on the Illumina HiSeq 2500 platform. After the adaptors were removed and low quality reads from raw data were filtered, 6.22 Gb of raw paired-end reads was generated. A total of 116,922,726 cleaned reads were obtained from the four groups. Meanwhile, 77.92–78.84% reads and 60.74–61.76% unique reads were successfully aligned to the chicken reference genomes. The average Q30 value was higher than 94.33% (Table 6). These results indicated that the sequencing results were reliable enough for further analysis.

**Table 6:** Summary statistics for sequence quality and alignment information of 12 samples from duodenal mucosa^a^.

**Groups**	**Control**	**0.02% quercetin**	**0.04% quercetin**	**0.06% quercetin**
Clean reads	37,267,359	37,309,354	37,631,133	36,765,461
Q20 (%)^b^	98.28	98.26	98.33	98.16
Q30 (%)^c^	94.69	94.64	94.82	94.33
Total mapped reads	29,379,180	29,071,017	29,507,397	28,965,132
Uniquely mapped reads^d^	23,010,696	22,664,499	23,007,384	22,612,896
Multiple mapped reads	6,368,484	6,406,518	6,500,013	6,352,236
Total mapping ratio (%)^e^	78.84	77.92	78.41	78.78
Uniquely mapping ratio (%)^f^	61.76	60.74	61.14	61.50

a *n = 3 for all groups*.

b *Q20: The proportion of base number with a mass value >20 in reads after filtration accounted for the total base number*.

c *Q30: The proportion of base number with a mass value >30 in reads after filtration accounted for the total base number*.

d *Uniquely mapped reads = reads that matched only one position in the genome*.

e *Mapping ratio = mapped reads/clean reads*.

f *Unique mapping ratio = mapped unique reads/clean reads*.

### Differentially Expressed Genes of Quercetin in the Duodenum of Broilers

**Figure 1:**
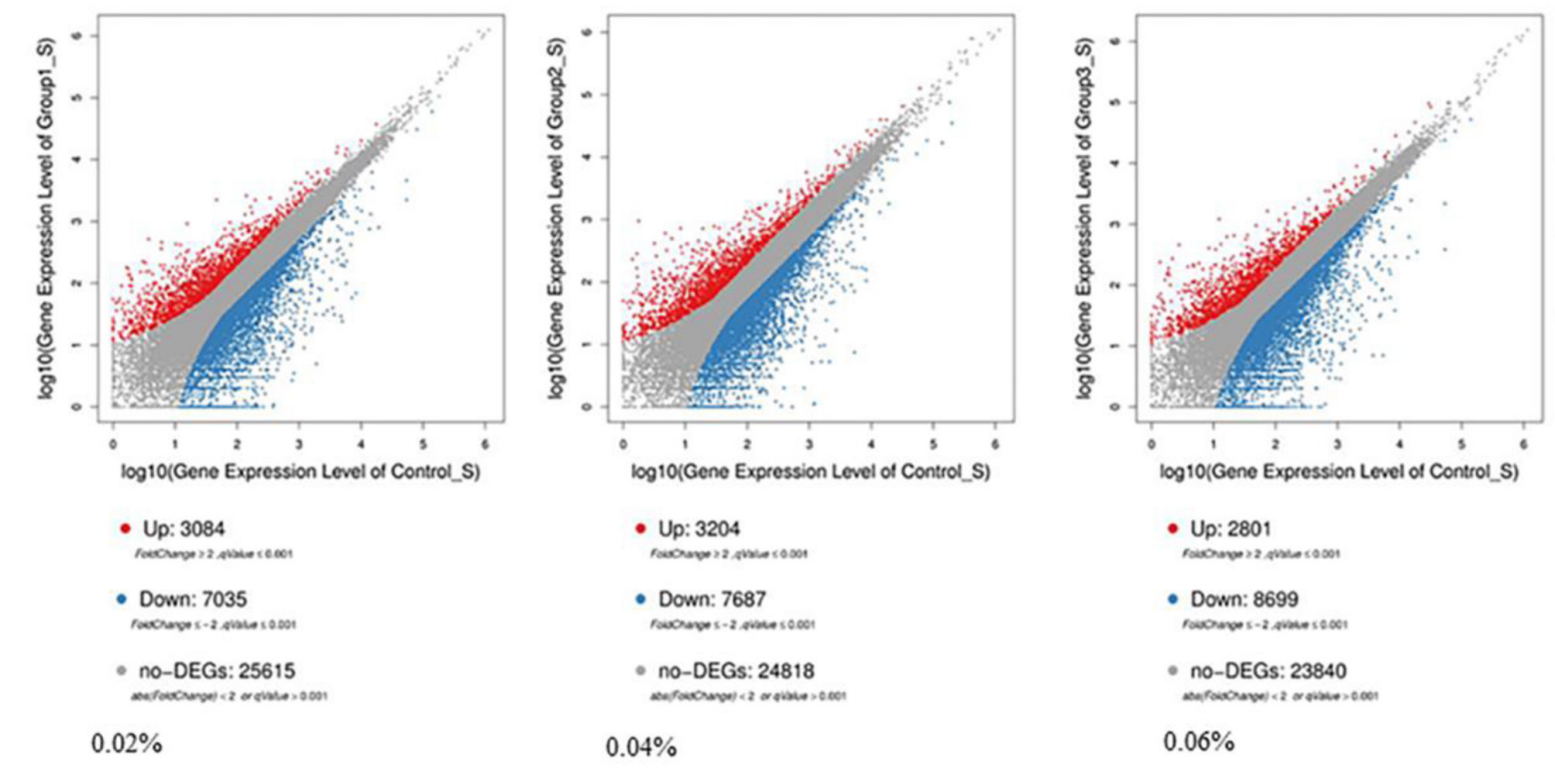
Comparison of differentially expressed genes between control and quercetin (0.02, 0.04, and 0.06%). The volcano plot shows correlations in the gene-rich dimension. The red and blue dots represent significantly upregulated and downregulated genes by quercetin, respectively.

Compared with control, 7,035 differentially expressed genes (DEGs) were downregulated and 3,084 DEGs were upregulated among 10,119 significant DEGs in the 0.02% quercetin group; 7,687 DEGs were downregulated and 3,204 DEGs were upregulated among 10,891 significant DEGs in the 0.04% quercetin group; And 8,699 DEGs were downregulated and 2,801 DEGs were upregulated among 11,500 significant DEGs in the 0.06% quercetin group (Figure 1).

### Functional Annotation and Signaling Pathway Enrichment of Differentially Expressed Genes

**Figure 2:**
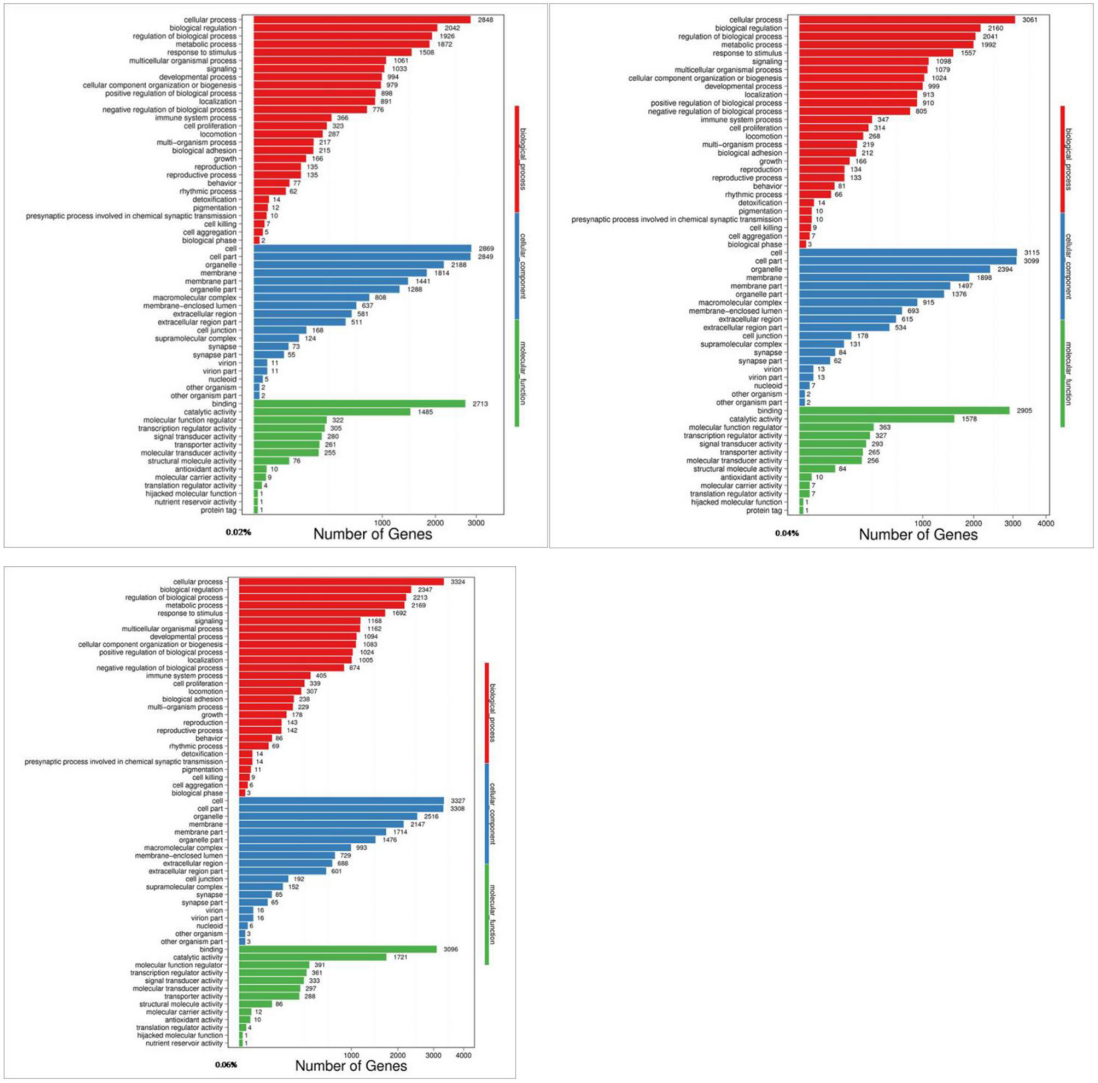
GO analyses of quercetin affecting differentially expressed genes in the duodenal mucosa of broilers (0.02, 0.04, and 0.06%).

The Gene Ontology (GO) knowledgebase is the largest source of information on the functions of genes in the world, which is divided into three parts, including biological process, cellular component, and molecular function. Among them, the metabolic process in biological processes ranked the fourth among the most abundant GO terms (Figure 2).

The present results showed that 9,231 DEGs of control/0.02% quercetin were annotated into 334 pathways, 9,899 DEGs of control/0.04% quercetin were annotated into 334 pathways, and 10,544 DEGs of control/0.06% quercetin were annotated into 337 pathways (Table 7).

**Table 7:** Important differentially expressed genes in the Wnt signaling pathway.

**Gene ID**	**Gene name**	${Log}_{2}^{FC}$ **^a^**	***q*-value^b^**	***P*-value^c^**	**Type**
**0.02% quercetin**					
XM_015292954.2	Wnt-5a	−3.56	1.36E^−35^	2.55E^−36^	Wingless-type MMTV integration site family, member 5
XM_015288320.2	CAMK2G	−7.53	1.49E^−21^	5.09E^−22^	Calmodulin-dependent protein kinase II
XM_015276289.2	CAMK2D	−5.68	1.41E^−07^	1.38E^−07^	Calmodulin-dependent protein kinase II
XM_025142761.1	CAMK2B	−1.48	7.44E^−05^	0.000105	Calmodulin-dependent protein kinase II
NM_001199435.1	PLCB4	−2.59	2.09E^−50^	2.51E^−51^	Phosphatidylinositol phospholipase C, beta
XM_025141605.1	PRKCA	8.93	8.09E^−47^	1.07E^−47^	Classical protein kinase C alpha type
XM_025147635.1	NFATC1	−4.83	7.52E^−05^	0.000106	Nuclear factor of activated T cells, cytoplasmic 1
**0.04% quercetin**					
XM_015292954.2	Wnt-5a	−3.88	2.40E^−38^	6.22E^−39^	Wingless-type MMTV integration site family, member 5
XM_015288320.2	CAMK2G	−7.57	6.61E^−22^	3.21E^−22^	Calmodulin-dependent protein kinase II
XM_015276289.2	CAMK2D	−5.71	9.31E^−08^	1.15E^−07^	Calmodulin-dependent protein kinase II
XM_025142761.1	CAMK2B	−1.19	0.000479	0.000934	Calmodulin-dependent protein kinase II
NM_001199435.1	PLCB4	−2.11	1.56E^−40^	3.77E^−41^	Phosphatidylinositol phospholipase C, beta
XM_025141605.1	PRKCA	–	–	–	Classical protein kinase C alpha type
XM_025147635.1	NFATC1	−4.87	5.51E^−05^	9.42E^−05^	Nuclear factor of activated T cells, cytoplasmic 1
**0.06% quercetin**					
XM_015292954.2	Wnt-5a	−4.89	3.58E^−42^	8.39E^−43^	Wingless-type MMTV integration site family, member 5
XM_015288320.2	CAMK2G	−7.53	9.98E^−22^	5.28E^−22^	Calmodulin-dependent protein kinase II
XM_015276289.2	CAMK2D	−5.67	1.01E^−07^	1.41E^−07^	Calmodulin-dependent protein kinase II
XM_025142761.1	CAMK2B	−1.62	1.82E^−05^	3.28E^−05^	Calmodulin-dependent protein kinase II
NM_001199435.1	PLCB4	−1.00	3.84E^−14^	3.15E^−14^	Phosphatidylinositol phospholipase C, beta
XM_025141605.1	PRKCA	5.60	2.23E^−07^	3.23E^−07^	Classical protein kinase C alpha type
XM_025147635.1	NFATC1	−4.84	5.57E-^05^	0.000107	Nuclear factor of activated T cells, cytoplasmic 1

a $Log_{2}^{FC}$*^(sample2/sample1)^: differential expression multiple between samples (groups) after log_2_ conversion*.

b *q-value: the corrected P-value. The smaller the q-value, the more significant the difference in gene expression*.

c *P-value: significant statistical value*.

**Figure 3:**
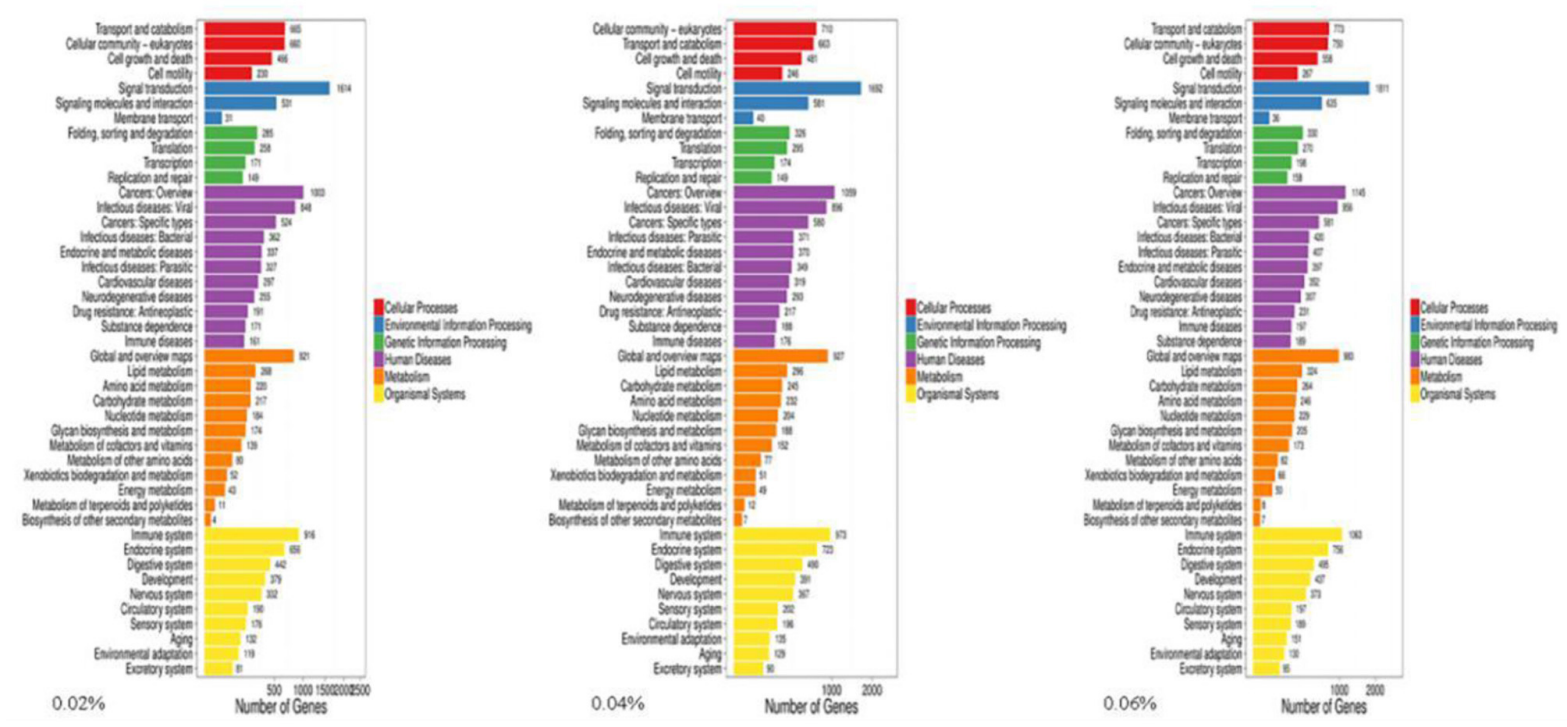
KEGG of differentially expressed genes in control and quercetin (0.02, 0.04, and 0.06%).

Kyoto Encyclopedia of Genes and Genomes (KEGG) enrichment analysis showed that the Wnt signaling pathway was the main signaling pathway of calcium and phosphorus metabolism in broilers, which was significantly regulated by quercetin. Signal transduction accounted for the largest proportion in KEGG analysis, and the Wnt signaling pathway was included in signal transduction (Figure 3). Among them, *q* ≤ 0.01 and |${\text{log}}_{2}^{\left(\text{fold change}\right)}$|≥1 were selected as the conditions for filtering DEGs. The main DEGs in the Wnt signaling pathway were as follows: Wnt family member 5a (Wnt-5a), calcium/calmodulin-dependent protein kinase II (CAMK2G, CAMK2D, CAMK2B), phospholipase C, beta 4 (PLCB4), protein kinase C alpha (PRKCA), and nuclear factor of activated T cells-1 (NFATC1) (Table 8).

**Table 8:** Summary of DEGs involved in calcium and phosphorus metabolism.

**Gene ID**	**Gene name**	${Log}_{2}^{FC}$			**Type**
		**0.02% quercetin**	**0.04% quercetin**	**0.06% quercetin**	
XM_015292954.2	Wnt-5a	−3.56	−3.88	−4.89	Wnt signaling pathway
XM_015288320.2	CAMK2G	−7.53	−7.57	−7.53	Wnt signaling pathway
XM_015276289.2	CAMK2D	−5.68	−5.71	−5.67	Wnt signaling pathway
XM_025142761.1	CAMK2B	−1.48	−1.19	−1.62	Wnt signaling pathway
NM_001199435.1	PLCB4	−2.59	−2.11	−1.00	Wnt signaling pathway
XM_025141605.1	PRKCA	8.93	–	5.60	Wnt signaling pathway
XM_025147635.1	NFATC1	−4.83	−4.87	−4.84	Wnt signaling pathway

*$Log_{2}^{FC}$ ^(sample2/sample1)^: differential expression multiple between samples (groups) after log_2_ conversion*.

### Validation of the Wnt Signaling Pathway by RT-qPCR

**Figure 4:**
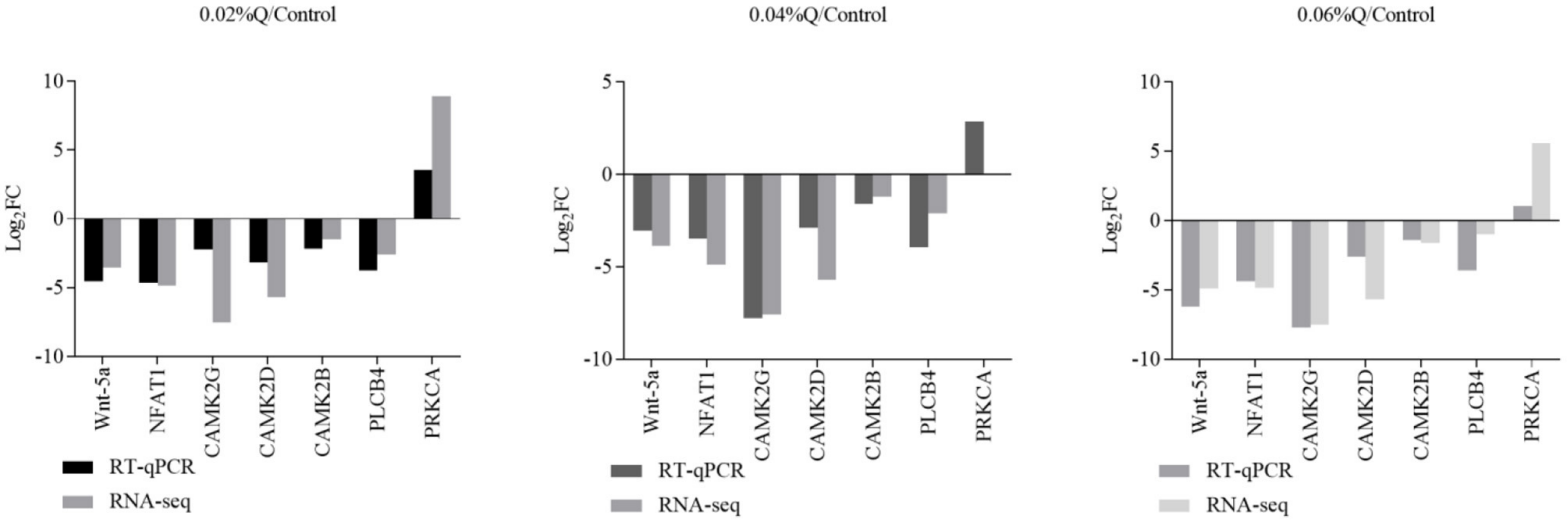
Correlations of mRNA expression level of seven random DEGs between the level of calcium and phosphorus using RNA-seq and RT-qPCR. The X-axis represents the seven selected genes for RT-qPCR assays and the Y-axis represents the ${\text{log}}_{2}^{\left(\text{fold change}\right)}$ derived from RNA-seq. Values are mean ± SEM (*n* = 6). 0.02, 0.04, and 0.06%Q represent 0.02% quercetin, 0.04% quercetin, and 0.06% quercetin, respectively.

**Figure 5:**
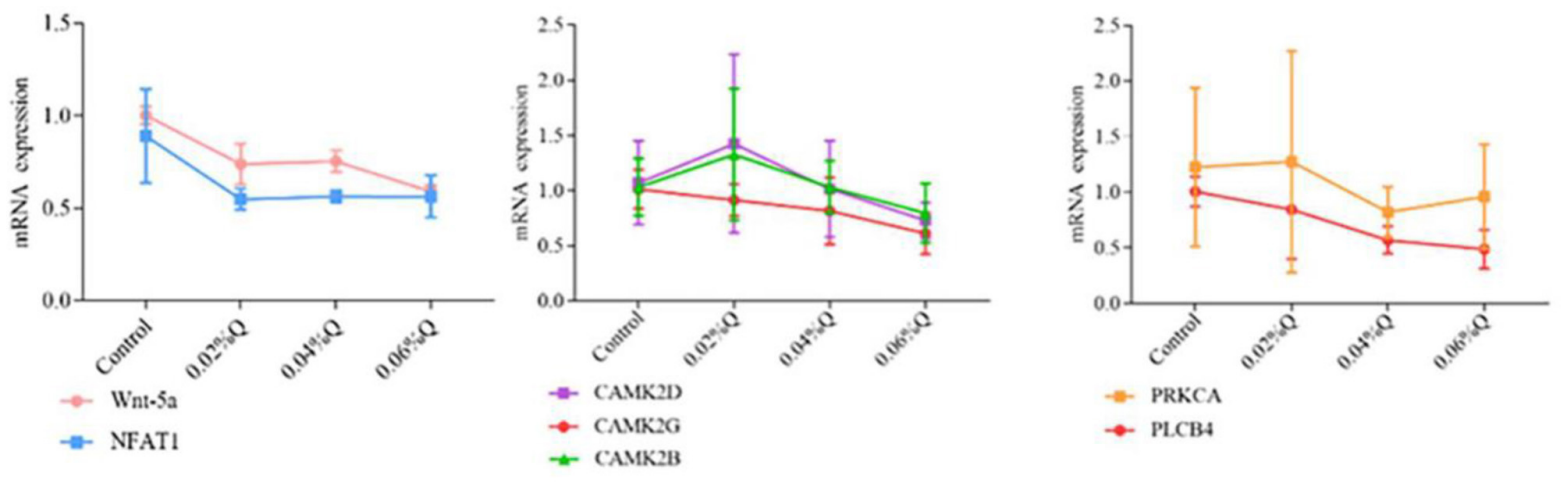
Effect of quercetin on mRNA expression of genes related to calcium and phosphorus metabolism in duodenal mucosa. (1) The results of relative quantification are expressed as 2^−ΔΔCT^. The quantification of control is 1, namely 2^−ΔΔCT^ = 1. The value 2^−ΔΔCT^ of the treatment group is a multiple of control. *n* = 6. (2) Mean values without a common letter are significantly different, *P* < 0.05. Values are mean ± SEM (*n* = 6). 0.02, 0.04, and 0.06%Q represent 0.02% quercetin, 0.04% quercetin, and 0.06% quercetin, respectively.

To verify the accuracy of the RNA-seq results in the transcriptome, the main DEGs in the Wnt signaling pathway were selected as follows: Wnt-5a, NFATC1, CAMK2G, CAMK2D, CAMK2B, PLCB4, and PRKCA, and β-actin was used as a house-keeping gene. The results showed that the mRNA expression by RT-qPCR was consistent with the transcriptome RNA-seq results, it indicated that the sequencing results were reliable (Figures 4, 5).

## Discussion

Quercetin is the major representative of plant flavonoids, which ubiquitously found in fruits, kales, and cherries, as well as onions and red wine, is one of the most common flavonoids in the diet (29–31). Modern research has shown that quercetin may prevent various diseases, such as osteoporosis (32), tumors (33), and lung diseases (34). At present, there are no reports about quercetin preventing TD and regulating calcium and phosphorus metabolism. Induced TD diet combined with cholecalciferol at 1,500 IU/kg improved tibia ash, performance, and prevented TD in 1-day-old broilers (35). BMD, bone weight index, tended to increase with dietary quercetin (100 mg/kg/day) supplementation in rats (*P* < 0.05) (36). Diet supplemented with 2.50% quercetin increased bone density in ovariectomized rats (*P* < 0.05) (37). In addition, icariin prevented TD in broiler chickens, tibia weight was increased with the increasing icariin, and icariin inhibited bone resorption and stabilized bone bioapatite, it indicated that the BMD of growing rats supplemented with total flavone capsules from icariin was significantly increased (*P* < 0.05) (38). Meanwhile, compared with control, the tibia length of the low genistein group was longer (*P* < 0.05), and a high genistein level may stimulate the subchondral bone of the mandible, thereby it is beneficial to bone formation in rats (39, 40). Moreover, in the heat neutral and heat stress groups, supplementation of 25-hydroxycholecalciferol (25-OH-D_3_) and soy isoflavones linearly increased BMD, content of ash, Ca, and P in tibia (*P* < 0.05) (28). Our results showed that dietary supplementation of quercetin at the level of 0.06% significantly increased relative weight, unit weight, P, BMD, and ash of tibia (*P* < 0.05). The present results were supported by previous studies on the effect of flavonoids on bone development and calcium and phosphorus deposition in broilers (41).

To further study the development of the tibia and the deposition of Ca and P in the tibia of broilers, the serum biochemical indicators of broilers were determined and a duodenal transcriptomic study was carried out. Calcium provides the power to support movement for bone, however, it is also a reservoir for maintaining serum calcium levels (42, 43). Calcium metabolism is achieved by bone, kidney, and intestine, and intestinal calcium metabolism is the key. Calcium transport in the intestine is regulated by a complex homeostatic hormone system, mainly including 1,25-(OH)_2_D_3_ and serum Ca (44). Among them, 1,25-(OH)_2_D_3_ is one of the important hormones regulating bone metabolism. 1,25-(OH)_2_D_3_ reaches the intestine and bone tissue through the blood, and binds to specific receptors in the small intestinal mucosa, thereby increasing the synthesis of calcium-binding protein, which in turn promotes the absorption of Ca^2+^ and the process of bone calcification (45). Furthermore, soy isoflavone supplementation significantly improved levels of serum OC, vitamin D, Ca, P, and ALP activity in quail at the later laying stage (46). In addition, dietary supplementation of daidzein and Chinese herbs (CH) at the level of 0.02% significantly increased the content of serum Ca, P, and OC in laying hens (47). Similarly, in laying hens, serum calcium was linearly increased with the addition of daidzein, and serum phosphorus had a significantly conic response to the addition of daidzein (48). Compared with control, dietary supplementation of genistein (400 mg/kg) increased the content of serum CT and ALP, which was consistent with the increase in levels of calcium and phosphorus in the tibia of menopausal women (15). E_2_ may upregulate the expression of ATP-dependent Ca pump in the human uterine plasma membrane (49). Therefore, the present results showed that dietary supplementation of quercetin at the level of 0.06% significantly increased the content of serum E_2_, OC, CB, ALP, and CT (*P* < 0.05), and decreased the content of serum PTH (*P* < 0.05) in broilers. Results of serum ALP in our experiment were supported by Chen et al. who found that alfalfa flavonoids extraction increased serum ALP level in Yangzhou geese (50).

Consistently, it is important to study the site of calcium absorption in the body. Intestinal Ca^2+^ absorption is an active (ATP-dependent) process, which mainly occurs in the small intestine, accounting for about 90% of Ca^2+^ absorption (51). The absorption of Ca^2+^ in the intestine mainly depends on the staying time in the intestine and the solubility of Ca^2+^. However, the duodenum is the site with the greatest solubility of Ca^2+^ (52). The rate of Ca^2+^ absorption in the intestine is as follows: duodenum > jejunum > ileum (53). Moreover, Ca^2+^ is absorbed through two pathways in the intestine: the transcellular process which primarily comes up in the duodenum and is mediated by vitamin D, and the paracellular concentration-dependent diffusional process which occurs throughout the intestine (54). Duodenal mucosa is the best site to explore the mechanism of quercetin regulating calcium and phosphorus metabolism. Hence, transcriptomic techniques were used to explore the effect of quercetin on calcium and phosphorus metabolism in the duodenal mucosa of broilers.

RNA-seq is an important means to study gene expression, RNA biosynthesis, and metabolism. Most importantly, RNA-seq directly reveals sequence consistency, which is essential for analyzing quantitative gene expression and investigating detailed transcriptomic profiles (55, 56). RNA-seq may obtain the full-length transcriptome sequence in animals, which has been widely used in studying the regulatory mechanism of metabolism and gene expression in livestock and poultry (57). RNA-seq has been applied in the heart (58), uterus (59), and ovarian tissues in chickens (60), and also revealed that quercetin regulated calcium absorption (61). Nonetheless, the precise mechanism of quercetin regulating calcium and phosphorus metabolism in the duodenum of broilers is still unclear. In this study, signaling pathways related to calcium and phosphorus metabolism were determined using RNA-seq, and the results were more accurate and effective of differential gene expression from high-quality sequencing and accurate mapping reading ratios (Table 6).

After functional enrichment analyses, most GO terms and KEGG pathways were mainly involved in metabolism processes. The results were similar to previous studies in cattle and pigs (62, 63), and implied that all identified DEGs may take part in calcium and phosphorus metabolism in the duodenum. The Wnt signaling pathway was highly correlated with calcium and phosphorus metabolism, and seven important genes of Wnt-5a, CAMK2G, CAMK2D, CAMK2B, PLCB4, PRKCA, and NFATC1 in regulating calcium and phosphorus absorption and metabolism were screened out. Meanwhile, to validate the results of RNA-seq, expression of the genes related to the calcium and phosphorus metabolism pathway were determined using RT-qPCR. Overall, RT-qPCR results were highly consistent with RNA-seq results, which were similar to some results previously obtained in rats (64) and chickens (65, 66).

The Wnt signaling pathway played a critical role in normal skeletal homeostasis and function. Wnt signaling cascades may play a centrally regulatory part in the development of calcium signal (67). The binding of Wnt-5a to the homologous Frizzled (Fz) receptor temporarily increases the concentration of inositol 1,4,5-triphosphate (IP3), Ca^2+^, and 1,2-diacylglycerol (DAG) intracellular signaling molecules. IP3 and DAG were derived from phospholipase C in the plasma membrane. Then IP3 underwent diffusion in the endoplasmic reticulum (ER) membrane and interacted with calcium channels, this process released Ca^2+^, then Ca^2+^ and calmodulin activate calmodulin-independent protein kinase II (CaMKII), and Ca^2+^ activates protein kinase C (PRK) through releasing ER (68–70). In this study, compared with control, quercetin supplementation significantly regulated the key genes of calcium and phosphorus metabolism in the Wnt signaling pathway. Wnt-5a played a crucial role in the atypical Wnt/PCP and Wnt/PKC pathways of osteoarthritis osteoblasts (71). The concentration of Ca^2+^ required for the activation of CAMK2B, CAMK2D, and CAMK2G depends on calmodulin level in the reaction (72). In the regulation of bone metabolism, the calcineurin-NFAT pathway acted on osteoclasts, osteoblasts, and chondrocytes (73). NFATC1 was highly expressed in RANKL-induced osteoclasts. Subsequently, calcineurin dephosphorylated the serine residues in NFATC1, which then entered the nucleus to initiate osteoclast formation (74). It was consistent with our results that NFATC1 levels were significantly downregulated. Phosphatidylinositol-4,5-diphosphate was converted to diacylglycerol and inositol-1,4,5-triphosphate by the PLCB4 enzyme, which may promote the release of intracellular calcium and activate protein kinase C, and osteoclasts were profoundly changed (75). Wnt-5a, CAMK2G, CAMK2D, CAMK2B, PLCB4, PRKCA, and NFATCI were key transcription factors regulating calcium and phosphorus metabolism. Flavonoids may affect the key regulators of calcium and phosphorus metabolism in the Wnt signaling pathway. Studies found that quercetin may protect bone and inhibit osteoclast formation, which may be involved in the regulation of the Wnt signaling pathway (76). And apigenin (API) may enhance the expression of downstream target genes in the Wnt signaling pathway, thereby improving new bone formation and accelerating fracture healing *in vivo*, so API may be a promising candidate for fracture treatment (77). Genistein maintained intracellular Ca^2+^ concentration by activating the CAMK signaling pathway (78). Neohesperidin prevented bone loss in ovariectomized mice by inhibiting NFAT (79). The anti-osteoclast activity of luteolin is mediated by blocking the activity of NFATc1, which may be a potential treatment for lytic bone diseases associated with osteoclast formation and dysfunction (80). Other results indicated that flavonoids improved tibia development and prevented osteoporosis by regulating genes related to calcium and phosphorus metabolism in the Wnt signaling pathway. Our results showed that diets supplemented with quercetin in broilers downregulated Wnt-5a, CAMK2B, CAMK2D, CAMK2G, PLCB4, and NFATC1 genes and upregulated the PRKCA gene. Therefore, quercetin used as a dietary additive potentially improved tibia development and balanced calcium and phosphorus metabolism.

## Conclusion

The present results showed that dietary quercetin supplementation improved calcium and phosphorus metabolism and tibia development by the Wnt signaling pathway. Quercetin was a potential functional additive preventing leg disease in broilers. Next, we will study the effect of quercetin in diseased broilers (lameness).

## Data Availability Statement

The datasets presented in this study can be found in online repositories. The names of the repository/repositories and accession number(s) can be found below: http://www.ncbi.nlm.nih.gov/sra/PRJNA781819, PRJNA781819.

## Ethics Statement

The animal study was reviewed and approved by the HEI Animal Management Certificate No. 11928.

## Author Contributions

BW designed the study and critically revised the first manuscript. Broilers were raised by BW, SW, and MD. HL and HW performed the experiments and participated in the statistical analysis. YL modified the manuscript and gave final approval of the version to be submitted. All authors agreed to be accountable for the content of the work and approved the submitted version.

## Funding

This work was supported by the National Natural Science Foundation of China (32072749).

## Conflict of Interest

The authors declare that the research was conducted in the absence of any commercial or financial relationships that could be construed as a potential conflict of interest.

## Publisher's Note

All claims expressed in this article are solely those of the authors and do not necessarily represent those of their affiliated organizations, or those of the publisher, the editors and the reviewers. Any product that may be evaluated in this article, or claim that may be made by its manufacturer, is not guaranteed or endorsed by the publisher.
